# Association of 25-hydroxyvitamin D deficiency with NT-pro BNP levels in patients with acute myocardial infarction: a cross-sectional analysis

**DOI:** 10.1186/1756-0500-4-542

**Published:** 2011-12-15

**Authors:** James B Wetmore, Rajyalakshmi Gadi, John H Lee, James H O'Keefe, Paul S Chan, Fengming Tang, John A Spertus

**Affiliations:** 1 Division of Nephrology and Hypertension, University of Kansas Medical Center, Kansas City, KS, USA; 2 University of Kansas Medical Center, Kidney Institute, Kansas City, KS, USA; 3 University of Kansas Medical Center, MS 3002, 3901 Rainbow Blvd, Kansas City, KS 66160, USA; 4 Department of Cardiovascular Outcomes Research, Saint Luke's Mid America Heart Institute, Kansas City, MO, USA

**Keywords:** Vitamin D, N-terminal proBNP, Acute myocardial infarction

## Abstract

**Background:**

Nutritional vitamin D deficiency is an emerging risk factor for acute myocardial infarction (AMI) and heart failure. The association of 25-hydroxyvitamin D levels with N-terminal pro B-type natriuretic peptide (NT-proBNP), a robust prognostic marker for post-AMI mortality and heart failure, is unknown and could illuminate a potential pathway for adverse outcomes among post-AMI patients with 25-hydroxyvitamin D deficiency.

**Methods:**

In a cross-sectional analysis, we studied 238 AMI patients from 21 U.S. centers to test the association of nutritional vitamin D (25-hydroxyvitamin D [25(OH)D]) deficiency with NT-proBNP levels. Levels of 25(OH)D levels were categorized as normal (≥30 ng/mL), insufficient (>20 - <30 ng/mL), deficient (>10 - ≤20 ng/mL), or severely deficient (≤10 ng/mL).

**Results:**

Low 25(OH)D levels were found in 95.7% of AMI patients. No significant trends for higher mean baseline log NT-proBNP levels in severely deficient (6.9 ± 1.3 pg/mL), deficient (6.9 ± 1.2 pg/mL), and insufficient (6.9 ± 0.9 pg/ml) groups were observed as compared with patients having normal (6.1 ± 1.7 pg/mL) levels, *P *= 0.17. Findings were similar in the subset of patients who had follow-up NT-proBNP levels drawn at one month. In multivariate regression modeling, after adjusting for multiple covariates, 25(OH)D was not associated with NT-proBNP.

**Conclusions:**

Potential associations between nutritional vitamin D deficiency and prognosis in the setting of AMI are unlikely to be mediated through NT-proBNP pathways. Future studies should examine other mechanisms, such as inflammation and vascular calcification, by which 25(OH)D deficiency could mediate adverse outcomes post-AMI.

## Background

Deficiency in nutritional vitamin D [25-hydroxyvitamin D, or 25(OH)D] is highly prevalent, occurring in approximately 30%-50% of the general population [1,2] and in an even greater percentage of hospitalized patients [3]. In several studies, 25(OH)D deficiency has been independently associated with both incident acute myocardial infarction (AMI) and heart failure (HF) [4,5], suggesting that 25(OH)D plays an important role in cardiac function. In support of this hypothesis, several in vitro studies have shown that calcitriol (1,25(OH)_2_D_3_), an active form of vitamin D, regulates intracellular calcium metabolism and myocardial contractility through specific vitamin D receptors on cardiac myocytes [5-8]. Consequently, 25(OH)D deficiency has been associated with aberrant cardiac contractility, cardiomegaly, and increased ventricular mass due to myocardial collagen deposition [6,7], independent of its known effects on blood pressure [6]. In studies of experimental animals, activation of nuclear vitamin D receptors by 1,25(OH)_2_D_3 _suppresses the expression and secretion of atrial natriuretic peptide (ANP) and brain natriuretic peptide (BNP) in cardiac myocytes [9-13], while in clinical studies of nondialysis chronic kidney disease patients, individuals treated with 1,25(OH)_2_D_3 _for 12 weeks were observed to have improved left ventricular diastolic function as compared with placebo [14]. Collectively, these findings suggest that low circulating levels of 25(OH)D could contribute to, or potentiate, the development of left ventricular dysfunction and HF after AMI.

In recent years, N-terminal pro B-type natriuretic peptide (NT-proBNP), a prohormone of BNP released from cardiac ventricles, has been associated with the severity of left ventricular dilatation and dysfunction after AMI [15,16]. In addition, NT-proBNP is a sensitive and robust prognostic biomarker of mortality in AMI [15,17-19], HF [20,21] and chronic hemodialysis patients [22,23]. Moreover, NT-proBNP levels have direct clinical implications, as they are used to guide therapeutic interventions in HF patients and lead to improved outcomes as compared with routine clinical treatment [24].

While both 25(OH)D and NT-proBNP are known to be associated with LV dysfunction after AMI, it is not known whether there is a correlation between levels of 25(OH)D and NT-proBNP in this setting. In clinical studies, inverse associations between 25(OH)D levels and NT-proANP in HF [5] and between 25(OH)D and BNP in dialysis patients have been reported [25,26]. However, these studies had small sample sizes and were not based on patients living in the U.S., where better nutrition and fortification of milk is common [27]. Given the adverse health implications of 25(OH)D deficiency and the lack of studies in AMI patients (a particularly high-risk group), we sought to examine the association of 25(OH)D deficiency with NT-proBNP levels in a multicenter cohort of AMI patients. Discovery of an association between circulating levels of 25(OH)D and NT-proBNP would not only suggest a potential pathway for adverse outcomes among post-AMI patients with 25(OH)D deficiency, but could also identify a potentially novel therapeutic target (i.e., nutritional vitamin D supplementation) to reduce NT-proBNP levels, in the hopes of improving prognosis after AMI.

## Methods

### Study participants

The present study is a cross-sectional analysis of a longitudinal cohort study. Participants were drawn from the TRIUMPH (Translational Research Investigating Underlying disparities in recovery from acute Myocardial infarction: Patients' Health status) study, a prospective multicenter cohort study of AMI patients across 21 U.S. centers [28]. Patients were eligible for TRIUMPH if they were ≥ 18 years of age and had a diagnosis of AMI. AMI was diagnosed by the presence of either a CK-MB elevation greater than twice normal or a troponin-I elevation of >0.1 mg/ml within 24 h of arrival to the hospital, accompanied by a clinical presentation suggestive of an AMI (e.g., prolonged ischemic signs or chest pain symptoms, at least one EKG with ST-wave elevation or ST-wave depression in 2 or more consecutive leads, and no alternative explanation for the presence of elevated serum cardiac markers). Patients were excluded if they transferred to the participating hospital from another facility greater than 24 h after their original AMI presentation, if they refused or could not provide informed consent, or if they were receiving hospice care. For this study, we included the last 250 TRIUMPH patients, in each of whom 25(OH)D levels were assessed in addition to the standard data collected [29]. TRIUMPH complied with the principles of the Declarations of Helsinki and was approved by the institutional review board at the coordinating center, Mid America Heart Institute at St. Luke's Hospital (Kansas City, MO). Written informed consent was obtained from all participants.

### Data collection

During index hospitalization, trained data collectors performed a patient interview and detailed chart abstraction within 24-72 h of admission. Patient data at AMI presentation, including demographic features, socioeconomic status, comorbidities, severity of AMI (ST elevation present or not), Killip class, Rose dyspnea index, vital signs, and laboratory values were abstracted. The Rose dyspnea score is a validated self-reported question in AMI patients that is scored from 0-4, with higher scores indicating worse dyspnea [30]. Regional and seasonal data were collected, given their association with 25(OH)D levels [31,32]. Patients were classified by their residing states in to 5 U.S. geographic regions, namely northeast (NE), southeast (SE), southwest (SW), midwest (MW) and west (W). Months of April-June, July-August and September-December were classified as summer, fall, and early winter seasons, respectively.

Left ventricular systolic function (LVSF) was classified as normal, mild, moderate or severe as assessed by echocardiography, angiography or nuclear imaging and documented in the hospital record. Finally, reperfusion therapy (percutaneous intervention [PCI] and/or coronary artery bypass surgery [CABG]) and other acute therapies, as well as medications prescribed at hospital discharge, were recorded. Blood samples from all consenting patients were sent to a core laboratory (Clinical Reference Laboratories, Lenexa, KS, USA) for NT-proBNP measurement (Roche Diagnostics, Indianapolis, IN, USA) and 25(OH)D assays, as described below.

### Laboratory measurements

An in vitro radioimmunoassay[33] assay (DiaSorin, Stillwater, MN, USA) was used for quantitative determination of 25(OH)D and other hydroxylated vitamin D metabolites in human serum. The DiaSorin 25(OH)D assay comprises a two-step procedure involving both a rapid extraction of 25(OH)D and other hydroxylated metabolites from serum or plasma with acetonitrile along with a 25(OH)D-specific antibody and tracer, and phase separation with a second antibody-precipitating complex. The total (intra-and inter-assay) precision of the assay has a coefficient of variation (CV) of 9.4% and 11% for control values of 8.6 ng/mL and 49.0 ng/ml, respectively.

For measurement of circulating NT-proBNP levels, the Roche electro-chemiluminescence immunoassay for the in vitro quantization was used (ECLIA Roche diagnostics, Mannheim, Germany). The precision of this assay was represented by a CV of 3.2% and 2.3% for control values of 175 pg/mL and 4962 pg/mL, respectively. Blood samples for NT-proBNP were drawn immediately prior to hospital discharge. The mean time of the NT-proBNP blood draw was 3.5 ± 4 days after enrollment. Glomerular filtration rate (eGFR) was estimated using the four variable Modification of Diet in Renal Disease (MDRD) study equation [34].

### Statistical analysis

Participants were classified into clinically-relevant categories on the basis of 25(OH)D levels. Patients with levels ≥30 ng/mL were classified as normal, while levels >20 ng/mL and <30 ng/ml were considered insufficient, levels of >10 to ≤20 ng/ml were deficient, and levels ≤10 ng/mL were severely deficient. Median NT-proBNP levels were compared using the non-parametric Kruskal-Wallis test, due to the positively-skewed distribution of NT-proBNP. Log NT-proBNP and quartiles of NT-proBNP were compared across the four 25(OH)D strata using ANOVA and chi-square tests, respectively, both at baseline and, in a complementary analysis, in the subgroup of patients who had 1-month levels of NT-proBNP levels drawn. To confirm previously-reported relationships, log NT-proBNP and category of LVSF was analyzed with the linear trend test. Spearman rank correlation was obtained between NT-proBNP and 25(OH)D levels. Multivariable linear regression analysis to determine the association of 25(OH)D levels with log NT-proBNP was performed, adjusting for relevant covariates and confounders. The presence of a potential interaction between NT-proBNP levels and type of AMI (i.e., ST-elevation versus non-ST-elevation) was formally tested using the likelihood ratio test. A *P-*value of <0.05 was considered statistically significant. All analyses were conducted using SAS v9.1 software (Cary, NC, U.S.).

## Results

Of the 238 enrolled patients on whom sufficient data was available, the median 25(OH)D concentration was 16 ng/mL (interquartile range [IQR], 12-21 ng/mL). As noted previously [29], classification of 25(OH)D levels into more clinically-interpretable ranges showed 40 (16.8%) to be severely deficient, 138 (57.9%) to be deficient, and 50 (21.0%) to be insufficient. Only 4.2% of participants in the study had normal 25(OH)D levels, with none of the African-American participants having normal levels.

Characteristics of participants across 25(OH)D groups are shown in Table 1. No statistically-significant differences by age or gender were noted across 25(OH)D groups. Deficiency in 25(OH)D was associated with non-Caucasian (i.e., African-American) race, lower socioeconomic status indicators, a history of recent smoking, and lower levels of physical activity. No difference in those with or without 25(OH)D deficiency were observed for diabetes, hypertension, chronic kidney disease, chronic HF, left ventricular hypertrophy on admission electrocardiogram, Kullip class, Rose dyspnea score, or the type of AMI (STEMI vs. NSTEMI). During the study period, no statistically-significant regional variations were observed in patients' enrollment across 25(OH)D groups, although there was a trend towards seasonal variation in 25(OH)D levels (*P *= 0.064), with the winter months being associated with the lowest levels. Estimated glomerular filtration rates did not differ. In terms of laboratory values, lower serum calcium and higher parathyroid hormone (PTH) levels were significantly associated with 25(OH)D deficiency (*P *= 0.03 and *P *= 0.004, respectively), as would be expected. Likewise, the use of omega-3 supplements was significantly greater higher in patients with normal 25(OH)D levels compared to those who had insufficient, deficient, or severely-deficient levels (*P *= 0.002).

**Table 1:** Baseline characteristics of study participants, by 25(OH)D group

		25(OH)D group, ng/mL			
	
	0-10	>10-≤20	>20-<30	≥30	
Characteristic	(*n *= 40)	(*n *= 138)	(*n *= 50)	(*n *= 10)	*P-*value
Demographics					
Age (yr)	55.0 ± 11.2	57.8 ± 11.8	58.8 ± 10.2	62.4 ± 11.3	0.21
Male, *n *(%)	26(65.0)	104(75.4)	38 (76.0)	8 (80.0)	0.57
Caucasian, *n *(%)	23 (57.5)	103 (74.6)	41 (82.0)	10 (100.0)	0.012
BMI (kg/m^2^)	32.1 ± 5.6	30.9 ± 6.9	29.5 ± 5.6	26.5 ± 4.6	0.066
Socio-economic status					
Low social support, *n *(%)	11 (27.5)	20 (14.9)	3 (6.0)	0 (0)	0.023
No health insurance, *n *(%)	14 (35.0)	28 (20.9)	5 (10.4)	1 (10.0)	0.036
Lifestyle					
Smoked within 1 month, *n *(%)	24 (60.0)	49 (35.5)	15 (30.0)	0(0)	<0.001
Leisure time activity, *n *(%)					0.047
Mainly sedentary	20 (50.0)	56 (40.9)	19 (38.0)	1 (10.0)	
Mild exercise	13 (32.5)	49 (35.8)	11 (22.0)	3 (30.0)	
Moderate exercise	6 (15.0)	27 (19.7)	17 (34.0)	4 (40.0)	
Strenuous exercise	1 (2.5)	5 (3.6)	3 (6.0)	2 (20.0)	
Geographical region, *n *(%)					0.79
Midwest	18 (56.3)	53 (45.3)	15 (34.1)	5 (62.5)	
Northeast	3 (9.4)	15 (12.8)	5 (11.4)	0 (0)	
Southeast	5 (15.6)	23 (19.7)	9 (20.5)	1 (12.5)	
Southwest	4 (12.5)	22 (18.8)	12 (27.3)	2 (25.0)	
West	2 (6.3)	4 (3.4)	3 (6.8)	0 (0)	
Season, *n *(%)					0.064
Apr-Jun	1 (2.5)	1 (0.7)	0 (0)	0 (0)	
Jul-Sep	10 (25.0)	56 (40.6)	24 (48.0)	7 (70.0)	
Oct-Dec	29 (72.5)	81 (58.7)	26 (52.0)	3 (30.0)	
Comorbidities, *n *(%)					
Diabetes	13 (32.5)	41 (29.7)	8 (16.0)	2 (20.0)	0.21
Hypertension	26 (65.0)	89 (64.5)	33 (66.0)	6 (60.0)	0.99
Prior MI	2 (5.0)	36 (26.1)	9 (18.0)	2 (20.0)	0.019
CKD	5 (12.5)	4 (2.9)	2 (4.0)	1 (10.0)	0.06
Chronic heart failure	3 (7.5)	6 (4.3)	1 (2.0)	1 (10.0)	0.38
LVH on admission ECG, *n *(%)	7 (17.9)	9 (6.7)	4 (8.2)	0 (0)	0.18
Killip class on arrival, *n *(%)					0.35
I	36 (94.7)	128 (94.8)	43 (86.0)	9 (90.0)	
II	2 (5.3)	4 (3.0)	5 (10.0)	1 (10.0)	
III	0 (0)	2 (1.5)	2 (4.0)	0 (0)	
IV	0 (0)	1 (0.7)	0 (0)	0 (0)	
Rose dyspnea score	1.0 ± 0.9	0.9 ± 0.9	0.8 ± 0.8	0.6 ± 1.0	0.43
Final MI diagnosis, *n *(%)					0.36
STEMI	15 (37.5)	64 (46.4)	23 (46.0)	2 (20.0)	
Non-STEMI	25 (62.5)	74 (53.6)	27 (54.0)	8 (80.0)	
LV systolic function, *n *(%)					0.54
Normal	24 (61.5)	88 (63.8)	36 (72.0)	6 (60.0)	
Mild	8 (20.5)	24 (17.4)	10 (20.0)	1 (10.0)	
Moderate	4 (10.3)	16(11.6)	4 (8.0)	1 (10.0)	
Severe	3 (7.7)	10 (7.2)	0 (0)	2 (20.0)	
Laboratory values					
Troponin I (ng/ml)	1.4 ±1.9	1.7 ± 1.8	1.6 ± 1.7	0.7 ± 1.2	0.3
Total Cholesterol (mg/dl)	150.6 ± 47	157.3 ± 29	150.5 ± 29	189.7 ± 47	0.011
HDL(mg/dl)	37.9 ± 10.6	38.4 ± 8.7	40.6 ± 10.7	43.1 ± 25.2	0.36
LDL (mg/dl)	92.2 ± 36.7	97.4 ± 25	90.4 ± 25.7	119.2 ± 42.8	0.032
Triglycerides(mg/dl)	153.2 ± 74	185.6 ± 145	143.6 ± 79	195.4 ± 169	0.17
Calcium (mg/dl)	8.9 ± 0.6	8.8 ± 0.6	9.0 ± 0.4	9.3 ± 0.6	0.038
Intact PTH (pg/ml)	59.8 ± 67.7	40.1 ± 28.1	32.8 ± 15.3	32.0 ± 26.5	0.004
Phosphate (mg/dl)	3.6 ± 0.7	3.5 ± 0.8	3.5 ± 0.7	3.7 ± 0.9	0.69
hs-CRP (mg/l)	5.2 ± 5.6	3.8 ± 5.3	3.8 ± 4.5	0.9 ± 0.8	0.12
eGFR, mL/min/1.73 m^2^	84.7 ± 28.1	88.3 ± 27.3	80.3 ± 18.9	80.1 ± 18.8	0.25
Medications					
ACEI/ARB, *n *(%)	11 (27.5)	39 (28.3)	18 (36.0)	1 (10.0)	0.43
Diuretic, *n *(%)	9 (22.5)	31 (22.5)	15 (30.0)	10(10.0)	0.58
Omega-3 supplements, *n *(%)	3 (7.5)	31 (22.5)	18 (36.0)	5 (50.0)	0.002

Continuous data shown as mean ± 1 standard deviation. 25(OH)D, 25-hydroxyvitamin D; BMI, body mass index; MI, myocardial infarction; CKD, chronic kidney disease; LVH, left ventricular hypertrophy; ECG, electrocardiogram; MI, myocardial infarction; STEMI, ST-segment elevation myocardial infarction; LV, left ventricular; HDL, high density lipoprotein; LDL, low density lipoprotein; PTH, parathyroid hormone; hs-CRP, high sensitivity C-reactive protein; eGFR, estimated glomerular filtration rate; ACEI, angiotensin converting enzyme inhibitor; ARB, angiotensin receptor blocker.

To confirm the existence of the expected association of LVSF with NT-proBNP levels, we examined this relationship in terms of both absolute and log-transformed baseline NT-proBNP levels. There was a significant graded association: individuals with normal LVSF had a mean baseline NT-proBNP level of 1296 pg/mL; those with mildly-decreased LVSF, 1833 pg/mL; those with moderately-decreased LVSF, 4461 pg/mL; and those with severely-decreased LVSF, 5776 pg/mL (*P *< 0.001 in the cases of both log-transformed and untransformed NT-proBNP levels).

**Figure 1:**
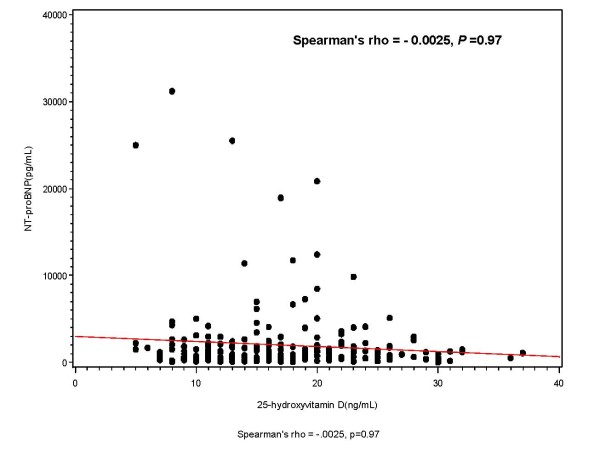
**Spearman correlation between levels of 25(OH)D and NT-proBNP**.

The association between baseline 25(OH)D and baseline log NT-proBNP levels are shown in Figure 1 andTable 2. No statistically-significant correlation between 25(OH)D and baseline log NT-proBNP levels was observed (rho = - 0.0025, *P *= 0.97; Figure 1). No significant trends for higher mean log NT-proBNP levels in severely deficient (6.9 ± 1.3 pg/mL), deficient (6.9 ± 1.2 pg/mL) and insufficient (6.9 ± 0.9 pg/mL) groups were observed as compared with patients having normal (6.1 ± 1.7 pg/ml) levels, (*P *= 0.17; Table 2). In a sensitivity analysis, when patients were stratified into quartiles of NT-proBNP, there was also no statistically significant association between 25(OH)D levels and category of NT-proBNP levels (*P *= 0.58, Table 2).

**Table 2:** Association of 25(OH)D levels with NT-proBNP levels

		25(OH)D level, ng/mL			
	
	0-10	>10-≤20	>20-<30	≥30	
Characteristic	*(n *= 40)	*(n *= 138)	*(n *= 50)	*(n *= 10)	*P-*value
Median NT-proBNP (pg/mL)	1094.5	897.5	1065.5	757.5	0.46
Mean log NT-pro BNP	6.9 ± 1.3	6.9 ± 1.2	6.9 ± 0.9	6.1 ± 1.7	0.17
NT-pro BNP (pg/mL), *n *(%)					0.58
Quartile 1 (5 to <488), 58 (24.3)	11 (27.5)	34 (24.6)	10 (20.0)	3 (30.0)	
Quartile 2 (488 to <924.5), 61(25.6)	8 (20.0)	38 (27.5)	13 (26.0)	2 (20.0)	
Quartile 3 (924.5 to <1882), 59 (24.7)	9 (22.5)	31 (22.5)	14 (28.0)	5 (50.0)	
Quartile 4 (1882 to 31230), 60 (25.2)	12 (30.0)	35 (25.4)	13 (26.0)	0(0)	

Continuous data shown as mean ± 1 standard deviation. 25(OH)D, 25-hydroxyvitamin D; NT-proBNP, N terminal pro brain natriuretic peptide

Because NT-proBNP levels can vary in the setting of AMI, we analyzed the subset (n = 66) of individuals who had NT-proBNP levels drawn one month after discharge. Again, there was no association between initial 25(OH)D levels and 1-month NT-proBNP levels (*P *= 0.82)

In the multivariable linear regression model (shown in Table 3), after adjustment for demographic (age, race, gender), anthropometric (BMI), socioeconomic (social support, medical insurance), lifestyle (smoking status), seasonal, comorbidity (diabetes and history of prior MI), treatment (in-hospital percutaneous coronary intervention [PCI] or coronary artery bypass graft [CABG]), laboratory (high sensitivity C-reactive protein [hs-CRP], estimated GFR) and medication (omega-3 supplement use) factors, 25(OH)D levels were not significantly associated with log NT-proBNP levels (*P *= 0.12). However, NT-proBNP levels were independently and strongly associated with age, race, hs-CRP, and eGFR.

**Table 3:** Multivariable regression analysis showing association of factors with NT-proBNP levels

Covariate	β coefficient	*P*-value
Age, per 10-yr increment	0.27	0.0006
Caucasian race	0.365	0.04
Male sex	-0.165	0.31
BMI, per 5 units kg/m^2 ^increment	-0.105	0.08
Lack of social support	-0.209	0.31
No health insurance	0.26	0.19
Smoked within 1 month	0.03	0.85
Season, Nov-Feb vs. Mar-Oct	0.184	0.22
History of diabetes	-0.02	0.9
History of prior MI	0.262	0.12
In-hospital PCI	0.202	0.23
In-hospital CABG	-0.147	0.56
hs-CRP, per 5 units mg/L increment	0.449	<0.0001
eGFR, per 5 units mL/min increment	-0.04	0.008
Omega-3 supplements	-0.224	0.18
25(OH)D, per 10 ng/mL increment	-0.203	0.12

BMI, body mass index; MI, myocardial infarction; PCI, percutaneous coronary intervention; CABG, coronary artery bypass graft; hs-CRP, high sensitivity C-reactive protein; eGFR, estimated glomerular filtration rate; 25(OH)D, 25-hydroxyvitamin D

Finally, we considered the theoretical possibility that our results might be affected by type of AMI (ST-elevation versus non-ST-elevation), so we formally tested for an interaction between type of AMI and NT-proBNP levels; none was found (*P *= 0.2)

## Conclusions

In this cross-sectional observational study, we found no evidence of an association between levels of 25(OH)D and NT-proBNP upon discharge, nor was there evidence of an association at one month in the subset of tested patients. To our knowledge, this is the first study to examine levels 25(OH)D and NT-proBNP in an AMI population. Thus, while both 25(OH)D and NT-proBNP levels are associated with cardiovascular disease and heart failure, they appear to impact prognosis through different mechanisms in the setting of AMI. However, we extend our previous findings [29] in this AMI cohort by showing that 25(OH)D levels are associated with smoking, lower degrees of physical activity, and lesser use of omega-3 supplements in bivariate analysis, consistent with previous reports [1,31,35]. That low social support and lack of medical insurance were associated in a graded manner across fine categories of 25(OH)D level suggest that these factors, both of which are associated with higher morbidity and mortality after MI [36-38], should be investigated in more depth in future. Additionally, although our data do not support an independent association between 25(OH)D deficiency and a biomarker of left ventricular dysfunction, namely NT-proBNP, in the setting of AMI, the high prevalence of 25(OH)D deficiency, which has been associated with incident MI [4] in prior observational studies, is an important area of future investigation. It would be critical to examine, for instance, whether rectifying low nutritional vitamin D levels in post-AMI patients is associated with an improvement in long-term outcomes.

Our findings extend and confirm insights provided by Pilz et al., who studied patients with established CAD referred to coronary angiography [39]. While these investigators found that 25(OH)D deficiency was significantly associated with higher New York Heart Association functional HF classes in unadjusted analysis, this association was no longer significant after adjustment for other clinical variables. In contrast, our findings are somewhat dissimilar from prior observations in chronic hemodialysis patients, where lower 25(OH)D levels were found to be associated with significantly higher log BNP levels [25]. NT-proBNP is a more stable form of BNP and correlates well with BNP levels in HF patients [40]. Similar reports in chronic HF patients have also suggested an inverse, nonlinear association between 25(OH)D and NT-proANP levels [5], although a clinical trial of nutritional vitamin D supplementation failed to alter patients' NT-proBNP levels [41].

More recently, a large cohort of patients referred to coronary angiography in the LUdgwigshafen RIsk and Cardiovascular Health (LURIC) study suggested that 25(OH)D levels remained independently associated with NT-proBNP levels in a multivariable model (β = -0.180, *P*<0.001) [39]. It is unknown whether the discrepancy in our findings and those of previous studies is due to unmeasured confounding in prior reports, or to the impact of acute myocardial injury (present in all participants of our study) on NT-proBNP levels [42,43]. Acute effects of MI could overwhelm or mask the basal association of 25(OH)D with NT-proBNP found in a more stable condition (such as that of the participants of the LURIC study[39]), given that NT-proBNP levels are influenced by acute ischemia [33] and can change over time [44]. We did, however, specifically examine the association of 25(OH)D levels and 1-month NT-proBNP levels in a subset of patients and found results similar to our primary analysis of baseline (hospital discharge) NT-proBNP levels, suggesting that volatility in NT-proBNP levels in the setting of AMI may not be responsible for our findings.

The results of our study should be interpreted in the context of some limitations. Our sample size was small, and therefore was underpowered for some analyses. However, our study of 238 patients did have 80% power to observe an unadjusted correlation of 0.18 between 25(OH)D and NT-proBNP levels (a level comparable to that of the LURIC study[39]). Nevertheless, we cannot of course be entirely certain of our findings, so more work in this regard must be performed. Additionally, there were very few patients (n = 10) who had normal 25(OH)D levels, and if there is a non-linear association between outcome and normal 25(OH)D levels, we may have been underpowered to detect this. It is also possible that the association between the biomarkers we tested might have been stronger at a time of clinical stability or, conversely, at the time of acute presentation, since our samples for both 25(OH)D and NT-proBNP were taken prior to discharge; however the prognostic importance of NT-proBNP at the time of MI has consistently been shown to be strong [18,19], and we performed the 1-month analysis to increase confidence in our findings.

In conclusion, the mechanism by which nutritional vitamin D deficiency mediates outcomes in AMI patients does not appear to be through its effects on, or a relationship with, NT-proBNP. Future studies should better clarify the clinical mechanism by which 25(OH)D deficiency is associated with outcomes in AMI patients. Potential candidates might include more long-term processes such as inflammation and vascular calcification. While we did not observe an association between levels of 25(OH)D and NT-proBNP, we did find a remarkably high frequency of 25(OH)D deficiency among AMI patients, consistent with other reports of hospitalized patients [3]. Hospitalization for AMI offers clinicians an opportunity to not only identify modifiable risk factors and to optimize medications for secondary prevention, but also to address other comorbidities, such as 25(OH)D deficiency. Given that nutritional vitamin D is readily available, inexpensive, and has a good safety profile, future studies should investigate whether addressing 25(OH)D deficiency might improve outcomes in AMI patients, regardless of the mechanism of its known association with post-AMI risk.

## Abbreviations

25 (OH)D: 25-hydroxyvitamin D; AMI: Acute myocardial infarction; ANP: Atrial natriuretic peptide; BNP: Brain natriuretic peptide; CABG: Coronary artery bypass surgery; CAD: Coronary artery disease; eGFR: Estimated glomerular filtration rate; HF: Heart failure; HTN: Hypertension; hs-CRP: High sensitivity C-reactive protein; LVDF: Left ventricular dysfunction; MDRD: Modification of Diet in Renal Disease; NT-proBNP: N-terminal pro B-type natriuretic peptide; PCI: Percutaneous intervention; PTH: Parathyroid hormone levels; TRIUMPH: Translational Research Investigating Underlying disparities in recovery from acute Myocardial infarction: Patients' Health status; VSMC: Vascular smooth muscle cell.

## Competing interests

The authors declare that they have no competing interests.

## Authors' contributions

JBW participated in the analytic plan and wrote the final draft of the manuscript, incorporating all required revisions to earlier versions in response to reviewer critiques; RG conceived of the study design and analytical plan and drafted the manuscript; JHL reviewed and edited the manuscript; JHO participated in design of the study and reviewed the manuscript; PSC reviewed the design and statistical analysis; FT participated in the design and analyzed the data; and JAS participated in the overall design, coordination, and analytical plan of the study and reviewed and edited the manuscript. All authors read and approved the final manuscript.
